# CD8+ T-Cells in Juvenile-Onset SLE: From Pathogenesis to Comorbidities

**DOI:** 10.3389/fmed.2022.904435

**Published:** 2022-06-21

**Authors:** Coziana Ciurtin, Ines Pineda-Torra, Elizabeth C. Jury, George A. Robinson

**Affiliations:** ^1^Centre for Rheumatology Research, Division of Medicine, University College London, London, United Kingdom; ^2^Centre for Adolescent Rheumatology Versus Arthritis, Division of Medicine, University College London, London, United Kingdom; ^3^Centre for Cardiometabolic and Vascular Science, Division of Medicine, University College London, London, United Kingdom

**Keywords:** CD8+ T-cells, juvenile-onset SLE, immunopathology, comorbidities, atherosclerosis

## Abstract

Diagnosis of systemic lupus erythematosus (SLE) in childhood [juvenile-onset (J) SLE], results in a more severe disease phenotype including major organ involvement, increased organ damage, cardiovascular disease risk and mortality compared to adult-onset SLE. Investigating early disease course in these younger JSLE patients could allow for timely intervention to improve long-term prognosis. However, precise mechanisms of pathogenesis are yet to be elucidated. Recently, CD8+ T-cells have emerged as a key pathogenic immune subset in JSLE, which are increased in patients compared to healthy individuals and associated with more active disease and organ involvement over time. CD8+ T-cell subsets have also been used to predict disease prognosis in adult-onset SLE, supporting the importance of studying this cell population in SLE across age. Recently, single-cell approaches have allowed for more detailed analysis of immune subsets in JSLE, where type-I IFN-signatures have been identified in CD8+ T-cells expressing high levels of granzyme K. In addition, JSLE patients with an increased cardiometabolic risk have increased CD8+ T-cells with elevated type-I IFN-signaling, activation and apoptotic pathways associated with atherosclerosis. Here we review the current evidence surrounding CD8+ T-cell dysregulation in JSLE and therapeutic strategies that could be used to reduce CD8+ T-cell inflammation to improve disease prognosis.

## Introduction

Diagnosis of systemic lupus erythematosus (SLE) in childhood, juvenile-onset SLE (JSLE, onset <18 years of age, ~20% cases), presents a more severe disease phenotype with an increased cardiovascular disease (CVD) and standardized mortality risk (almost 6-fold) compared to adult-onset SLE (1–3) (Table 1). JSLE patients also have increased prevalence of renal and neuropsychiatric involvement compared to patients with adult-onset SLE (3). Together, this has resulted in reports of early and irreversible damage in JSLE patients, with one study reporting this in 44.2% of patients 3.8 years from diagnosis, commonly relating to kidney disease, scarring alopecia and cognitive impairment (13). It is speculated that these age-of-onset-specific differences in prognosis may be due to increased pro-inflammatory type-I IFN signatures in JSLE, where there have been reports of an 80–90% prevalence compared to around 50% seen in adult-onset patients (23).

**Table 1:** Summary of important demographic and clinical characteristics of patients with juvenile-onset compared to adult-onset SLE.

	**JSLE (onset <18)**	**Adult-SLE (onset ≥18)**	**References**
**Demographic**			
General prevalence	Around 1.9–25.7 (Worldwide) or 9.38–10.08 per 100,000 (US) or 15–20% of all SLE cases	Around 29–210 (Europe) or 48–367 per 100,000 (US)	(4–10)
Female predominance	Around 80%	Around 90%	(4, 8)
**Clinical outcomes**			
Standardized mortality ratio	18.3 standardized mortality ratio or 2-fold higher mortality rate vs. adult-SLE	3.1 standardized mortality ratio or 2-fold lower mortality rate vs. JSLE	(3, 11, 12)
Renal disease (nephritis)	44–60%	33–37.1%	(3, 7, 11–14)
Neuropsychiatric (central nervous system)	25–29%	19.6–20%	(3, 11)
Cardiovascular disease risk relative to healthy	100–300-fold	50-fold (women 35–44)	(1, 2, 15, 16)
**Therapy**			
Proportion on high dose prednisolone treatment	97%	82%	(12, 17)
Proportion on immunosuppressive therapy	66–68%	37–43%	(12, 17)
**Disease control**			
Remission off treatment	10.8–31%	14.5%	(18–22)
Remission on treatment	42–61%	29%	(18–22)
Low disease states	32–82%	37.2–44%	(18–22)

CVD is responsible for around 50% of all deaths in Western countries, and whilst this is a remarkable statistic alone, in JSLE the risk is exacerbated (1), and it is estimated that patients have a 100–300-fold increased risk of mortality from CVD compared to age-matched healthy individuals (2). This relative risk in JSLE is greater than in adult-SLE, where women with adult-SLE between the ages of 35–44 increases the risk of coronary artery disease by 50 times (15). Atherosclerosis, a chronic inflammation of the medium-sized to large arteries, secondary to lipid deposition within the sub-endothelial intimal layer (atherosclerotic plaque), is a major cause of this CVD. Investigating the early development of atherosclerosis in these younger, more severely affected JSLE patients is of great importance to improve long-term prognosis. The interplay between traditional CVD risk factors and factors associated with active disease, inflammation and steroid treatment could contribute to the early, accelerated atherosclerosis in JSLE patients (1, 24, 25). Whilst the precise mechanisms are yet to be fully elucidated, there is no doubt that atherosclerosis and JSLE share several autoimmune pathways, particularly regarding inflammation and dyslipidaemia (26).

**Figure 1:**
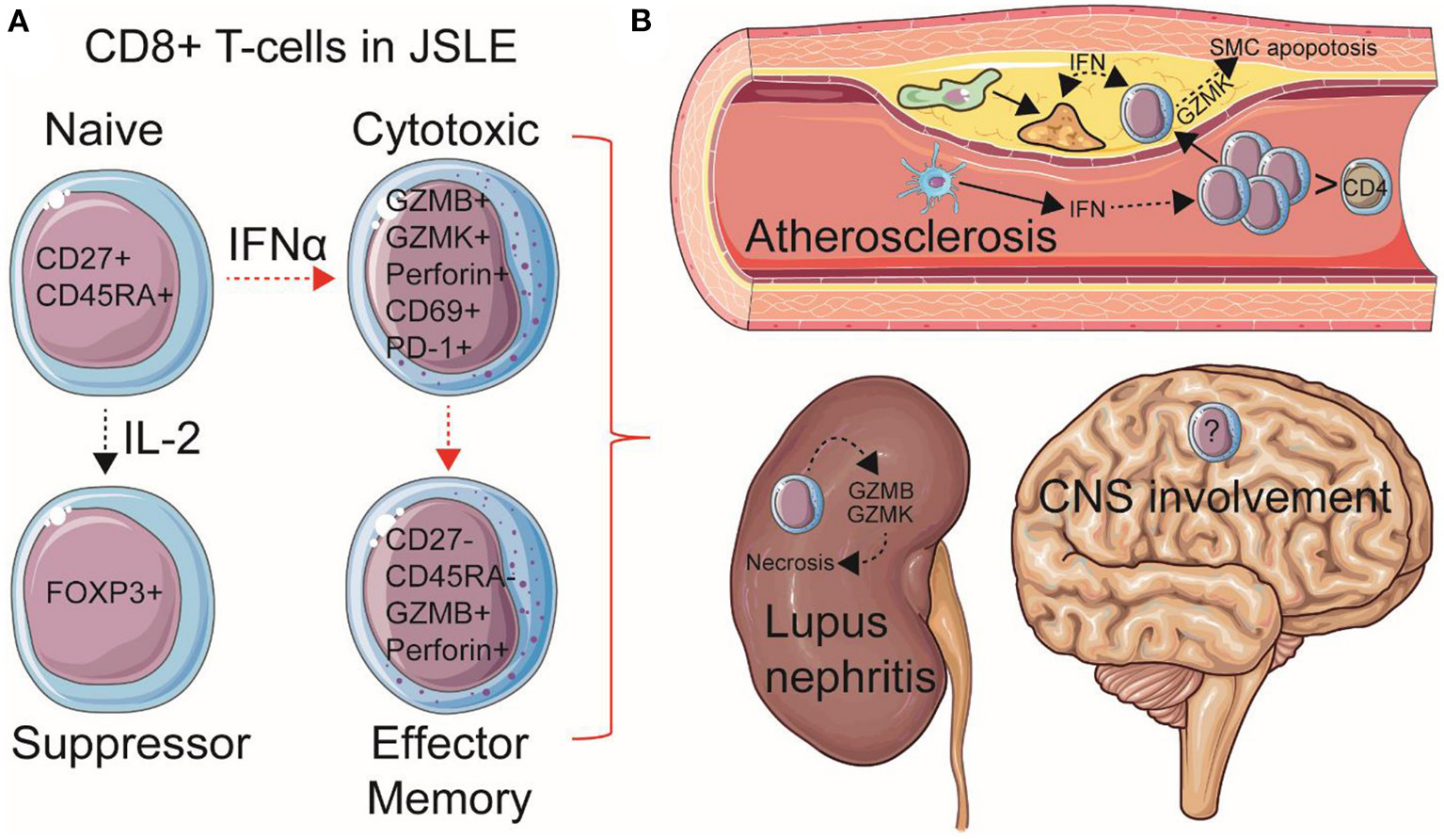
Summary of speculated pathogenic mechanisms of CD8+ T-cells in JSLE. **(A)** Total and naïve CD8+ T-cell frequencies are increased in circulation in JSLE patients. CD8+ T-cell functional differences have also been described in JSLE, including high expression levels of granzyme B/K (GZMB/K) and perforin transcripts, as well as CD69 and PD-1 expression, suggesting an activated, cytotoxic, pro-apoptotic, but exhausted CD8+ T cell profile. CD8+ FOXP3+ (suppressor) T-cells have also been described in JSLE using single cell technologies. Total and effector memory (EM) CD8+ T-cells have been associated with worse disease outcomes and organ involvement in JSLE, including **(B)** lupus nephritis and atherosclerosis (cardiovascular disease). Speculated mechanisms of organ involvement are displayed. With this respect, it has been recently shown that CD8+ T-cells are the predominant immune cell in established human atherosclerotic plaques and that high cardiometabolic risk JSLE patients have increased pro-inflammatory and pro-apoptotic circulating CD8+ T-cells associated with type-I interferon (IFN) signaling, which could influence plaque instability through effects on macrophage (green) foam cell (yellow) formation and apoptosis of vessel smooth muscle cells (SMCs). Dotted arrows represent indirect processes, including cellular maturation/differentiation and the effects of inflammatory mediators on cell signaling. Increased central nervous system (CNS) involvement is also associated with JSLE, however, immune mechanisms are less investigated. This Figure was produced using resources from Servier Medical Art, licensed under a Creative Common Attribution 3.0 Generic License. http://smart.servier.com/.

Despite these observations, few specific guidelines exist for the management of JSLE as an independent disease subset of SLE, especially as patients transition to adulthood, and basic research in this group of patients is scarce. There is an urgent need to investigate mechanisms of immunopathogenesis in this younger group of JSLE patients to improve disease monitoring, treatment options and quality-of-life. A recent abundance of findings surrounding a pathogenic role for CD8+ T-cells has emerged in JSLE research relating to their contribution to specific organ involvement and comorbidities such as atherosclerosis, as well as their systemic pro-inflammatory effects through functional and metabolic responses to type-I IFNs (Figure 1). Here we will review these new findings in JSLE and discuss possibilities for advancing therapeutic strategies to target this younger, more severely affected disease group.

## CD8+ T-Cells and the Pathogenesis of JSLE and Atherosclerosis

Due to greater focus on CD4+ T-cells in antibody-mediated autoimmunity (27, 28), there is a lack of CD8+ T-cell studies within the already scarce JSLE research available. In light of this, our recent study utilized sophisticated machine learning methodology to improve the analytical capacity of immune phenotype data collected from a JSLE patient cohort, where we investigated the immune profiles of adolescent JSLE patients compared to age-matched healthy individuals (HCs), as well as the longer-term clinical outcome measures from immune stratified groups (29). The study identified a unique and highly predictive immune signature of JSLE, validated using multiple machine learning methods, including a significant increase total and naïve CD8+ T-cells and reduced effector memory CD8+ T-cells, compared to HCs. Interestingly, total and effector memory CD8+ T-cells were significantly increased in 2 uniquely clustered patient groups that had a more active disease trajectory over 5-years of follow-up (increased average SLE Disease Activity Index, SLEDAI and decreased number of visits in Lupus Low Disease Activity State, LLDAS) and a higher prevalence of lupus nephritis (30). To our knowledge, this was the first time that CD8+ T-cells had been described and associated with pathogenic mechanisms and clinical outcomes in JSLE patients (Figure 1). Of note, CD4+ T-cells in this study were reduced in JSLE patients and were associated with better clinical outcomes, suggesting a more pathogenic role of CD8+ T-cells in JSLE. This key role of CD8+ T-cells has previously been identified in adult-onset SLE patients, where memory CD8+ T-cell expansion was associated with a worse prognosis of disease using transcriptional profiling (31, 32). Despite this, effector memory CD8+ T-cells in adult-onset SLE have been shown to have increased apoptotic profiles (increased tendency to undergo apoptosis upon stimulation) and decreased proliferative capacity, but also to express high levels of IFN-γ, perforin and granzyme B, highlighting possible pathogenic mechanisms of chronic inflammation and organ damage (33). In support, activated CD8+ T lymphocytes expressing perforin and granzyme B correlate positively with disease activity (SLEDAI) in adult patients (34, 35). RNA sequencing of lupus nephritis biopsy tissue in adult patients has also identified CD8+ T cells expressing high levels of *GZMB* and *GZMK* transcripts (36). Further investigations into the specific functional profiles of CD8+ T-cells in younger JSLE patients, their specific involvement in organ inflammation and damage, and how these compare to those of adult-SLE patients, will be important in addressing the increased damage and mortality risk in this patient population.

As well as improved analytical techniques, modern technologies have also enhanced the explorative capacity of specific immune cell population phenotypes in rare JSLE patient cohorts, as well as internal cohort heterogeneity, a strong reason for the failure of clinical trials (37). A recent study from Nehar-Belaid et al. has demonstrated this using single-cell RNA sequencing in a small cohort of 33 children with JSLE and 11 matched HCs (38). Despite detecting phenotype heterogeneity between patients, this study identified 2 unique T-cell sub-clusters of CD8+ T-cells that had strong transcriptomic cytotoxic programs (*PRF1, GZMB, GZMA*, and *KLRG1*), but unique helper T-cell profiles of either Th17 (*RORC* and *IL17RE*) or Th2/Tregs (*GATA3, CCR6* and *FOXP3*), compared to healthy individuals, supporting the complexity of CD8+ T-cell profiles in the context of JSLE. Studies in adult patients have explored similar CD8+ FOXP3+ (suppressor phenotype) T-cells in more detail, where these cells have been found to be defective or dysregulated, however, patient and cohort immune heterogeneity has resulted in mixed reports surrounding their frequency in circulation and functional phenotype in SLE compared to healthy individuals (39–41). Finally, in support of our previous study (30), this single cell analysis of JSLE also found that CD4+ T-cells were underrepresented in JSLE compared to healthy individuals, again supporting a more pathogenic role of CD8+ T-cells.

An important subset of CD8+ T-cells that should be considered in the context of JSLE are CD28- cells. Whilst most studies have focused on adult-SLE, it is speculated that these cells, with a tolerogenic and regulatory phenotype in healthy individuals, could have therapeutic potential in JSLE (42, 43). These cells have high antigen experience, resulting in downregulation of CD28 and subsequent shortening of telomeres through lack of telomerase activity, and therefore a senescent profile (long lived, terminally differentiated and oligoclonal) (44). This has led to controversy related to their anti- vs. pro-inflammatory role, with some studies reporting an upregulation of FOXP3 and Treg phenotype in healthy individuals (45–47), whilst others have reported cytotoxic markers such as granzyme A and perforin on their surface (48). This controversy has been further exacerbated in the context of SLE, where CD8+CD28- T-cells have been shown to lack FOXP3, positively correlate with disease activity (SLEDAI score) and be increased in patients with lupus nephritis (49). Instead of supporting a pro-inflammatory role for these cells, these observations could be the result of impaired anti-inflammatory functions in adult-SLE, as previously highlighted (50). In support of their anti-inflammatory potential, CD8+CD28- T-cells have also been shown to be increased in adult-SLE patients with inactive disease, whilst CD8+ CD28+ T-cells correlated positively with SLEDAI score and renal damage, (51). It is clear that additional markers are will be required to further categorize this subset of CD8+ T-cells (43), and further studies are warranted to uncover their pathogenic role in both JSLE and adult-SLE. In addition, their counterpart CD4+ CD28- T-cells have in fact a immunogenic and effector phenotype (52), highlighting the complexity of T-cell profiles and strength of single-cell technologies to improve targeted therapy toward distinct T-cell subsets in JSLE.

Cardiovascular disease represents one of the leading causes of mortality in JSLE through atherosclerosis (1). The vast majority of atherosclerosis research thus far has been focused on macrophages; however, the discovery of T-cell abundance in human atherosclerotic plaques has highlighted the participation of the adaptive immune system in atherogenesis (53). With this respect, CD8+ T-cells were recently shown to be extremely abundant in human atherosclerotic plaques (>30% of immune cells) (54). In this single-cell study, CD8+ T-cells were the most enriched immune subset in atherosclerotic plaques compared to peripheral blood (39 vs. 26%), whilst CD4+ T-cells were lower in frequency (50 vs. 65%), supporting active migration and a key pathogenic role of CD8+ T-cells. CD8+ T-cell cytotoxic functions can contribute to the promotion of cell death, necrotic core formation and plaque instability, where their relative frequency in plaques increases with progression (55, 56). Another single-cell study of human atherosclerosis identified a unique effector memory CD8+ T-cell subset in atherosclerotic plaques, expressing high levels of of *GZMK, GZMA*, and *CD69* (57), a similar profile to those seen upregulated in circulation in JSLE patients (30, 38). Enrichment of these pro-inflammatory circulating CD8+ T-cells in JSLE patients, highlights their possible association with the increased atherosclerotic risk for patients (Figure 1). In support, it has been shown recently by Robinson et al. that JSLE patients with an increased cardiometabolic risk, defined using biomarkers from adult SLE patients with sub-clinical atherosclerotic plaque, have a unique an exclusive increase in circulating CD8+ T-cells (58). These CD8+ T-cells from high cardiometabolic risk JSLE patients had increased activation and exhaustion markers (CD69 and PD-1), matching the previously described phenotype of CD8+ T-cells in human atherosclerotic plaques (54).

### Type-I IFN Signatures and CD8+ T-Cells in JSLE and Atherosclerosis; Implications for Treatment

Robinson et al. showed that CD8+ T-cells from high cardiometabolic risk JSLE patients had enriched activation, pro-apoptotic and SLE-specific IFN-signaling transcriptomic pathways associated with atherosclerosis, highlighted by a significant pathway enrichment overlap with transcriptomes of CD8+ T-cells isolated from human and mouse atherosclerotic plaques (54, 59). Importantly, altered IFN signatures and other shared pathways of atherosclerosis were not identified in matched isolated CD4+ T-cells in this study, suggesting that this is unique to the CD8+ T-cell compartment. As well as the increased prevalence of type-I IFN signatures in JSLE compared to adult-SLE patients (23), circulating type-I IFN levels are also associated with subclinical markers of atherosclerosis (60–62), including endothelial dysfunction and abnormal vascular repair (61, 63). Type-I IFNs have also been heavily implicated in multiple stages of the atherosclerotic process through driving pro-inflammatory responses (64–66). These include promoting immune cell recruitment and infiltration to arteries, subendothelial foam cell formation, fibrous cap thinning, plaque rupture and resultant thrombo-vascular events (67). The co-inflammatory role of type-I IFNs in T-cells in association with atherosclerosis is less described, however, it has been shown that pDCs colocalise with and stimulate T-cells in atherosclerotic plaques through IFNα, enabling cytotoxic T-cells to kill vascular smooth muscle cells, which can destabilize the plaque (66). In support, the same study showed that IFNα concentrations in atheroma tissues also correlated strongly with plaque instability scores. In addition, transcriptomic analysis of CD8+ T-cells isolated from atherosclerotic plaques has shown enriched IFN-signaling pathways as well as increased activation, exhaustion and cytotoxicity (54), supporting a link between IFNs, CD8+ T-cell activation and atherosclerosis.

Type-I IFN-signatures can now be identified at the single-cell level, allowing for more detailed analysis of cell subsets; in children with JSLE, a subset of CD8+ T-cells expressing high levels of *GZMK* transcripts have emerged with potent type-I IFN-signatures (38), representing 1 of the 3 immune subsets that were overrepresented in the JSLE group compared to HCs, and 1 of the 8 clusters (from 20) that contributed the most to the global IFN signature of JSLE patients. This supports a link between IFN and CD8+ T-cell function in JSLE that could be implicated in atherosclerosis. In contrast, type-I IFNs were recently shown to impact CD8+ T-cell metabolism in adult patients with SLE (68), where CD8+ T-cells from patients with a high IFN-signature had enlarged mitochondria and lower spare respiratory capacity associated with increased cell death due to prolonged IFN stimulation (62). Therefore, chronic stimulation of CD8+ T-cells with type-I could also reduce their frequency in JSLE long-term. As the investigations into the role of CD8+ T-cells in atherosclerosis expand, relating this to autoimmunity will be play a key part in understanding their pro-inflammatory role in exacerbating atherosclerosis progression.

Targeting CD8+ T-cells, either directly or through type-I IFN targeted therapies, could hold therapeutic potential in JSLE to reduce inflammation and disease-associated manifestations and co-morbidities such as nephritis and CVD (Figure 1). Current immunotherapies used in JSLE may not specifically alter CD8+ T-cells (69), as highlighted by our recent study, where we did not find major significant differences in conventional treatments between JSLE subgroups with high and low circulating CD8+ T-cells (30). However, new therapeutic strategies aimed at CD8+ T-cells could be beneficial.

With the shared anti-viral mechanisms (70) and mechanistic evidence discussed from studies in JSLE, there is a possibility for indirect therapeutic targeting of CD8+ T-cells via IFN pathways to reduce long-term damage and CVD from IFN-driven inflammation. With this respect, clinical trials blocking type-I IFN signaling through the IFN receptor or the JAK/STAT signaling pathway are already underway in SLE to reduce autoimmune inflammation (71, 72), which could also prevent atherosclerosis progression in patients. An example is the biologic anifrolumab, which has reached the primary endpoint [response by British Isles Lupus Assessment Group (BILAG) disease activity score] in an SLE trial (73). Anifrolumab binds to subunit 1 of the type I IFN receptor, blocking the activity of IFN-α, IFN-β and IFN-ω through the JAK/STAT pathway, thus preventing the expression of inflammatory genes. Janus kinase (JAK) inhibitors, licensed for use in inflammatory arthritis, act by inhibiting pro-inflammatory cytokine signaling through the JAK/STAT pathways (69) and they have been also tested in SLE and showed benefit in early phase trials (74, 75). Based on evidence highlighted by this review, stratifying patients by their inflammatory CD8+ T-cell profiles could improve the clinical efficacy of anifrolumab in the context of both long-term disease activity and cardiovascular outcomes. Patients in these clinical trials are already considered in terms of IFN gene signatures, and therefore, follow-up analysis of CD8+ T-cells pre- and post-treatment could highlight a key inflammatory pathway that is targeted (76). With this respect, blocking IFN receptors on the surface of CD8+ T-cells could reduce the cytotoxic profile induced by IFN to prevent both circulatory and tissue specific damage caused by pro-apoptotic mechanisms in JSLE.

Clinical trials in SLE do not usually include patients with JSLE, or stratify patients based on comorbidities such as dyslipidaemia, leading to mixed results regarding atherogenic lipid profiles and arterial thrombotic events in SLE (69), and therefore larger and better designed studies are warranted. Whether or not these CD8+ T cell targeted treatments will ultimately be beneficial for both JSLE and associated atherosclerosis will likely depend on the specific cell subsets, tissue penetration and JSLE phenotype specificity, disease activity and stage of atherosclerotic lesion progression.

### Additional CD8+ T-Cell Therapeutic Opportunity in JSLE

CD8+ T-cell targeted therapies for the future could involve inducing CD8+ suppressor T-cells in JSLE patients (77), especially as CD8+ suppressor T-cells have also been identified in atherosclerotic lesions of induced mouse models, where they carry out immunosuppressive functions and adoptive transfer of these cells reduces plaque size and macrophage infiltration (78). Low-dose IL-2 administration has already been shown to foster a dose-dependent increase in CD8+ suppressor T-cells in type-I diabetes patients (79), something that could be applied to JSLE patients for immunosuppression (80). It has also been shown that T-cell receptor stimulation with anti-CD3 monoclonal antibodies can specifically induce the CD8+ suppressor T-cell population, which could have a dual effect on JSLE activity and atherosclerosis progression. However, it has also been shown that CD8+ suppressor T-cells generated *in vitro* with IL-2 and GM-CSF from cells isolated from SLE patients with active disease or in remission, were not able to suppress in cells collected from patients with active disease, a function well-maintained by patients in remission (41). This suggests that these therapeutic strategies may depend on patient disease activity at the time of administration. It has also been shown that intravenous injection of a naturally occurring peptide, nucleosomal histone peptide H4 71–94, into lupus-prone mice can induce CD8+ suppressor T-cells, which can subsequently suppress lupus nephritis for up to 2 months (81). Cell-based immunotherapy using *in vitro* expanded CD8+ suppressor T-cells injected into mouse models engrafted with synovial tissues from patients with rheumatoid arthritis has also shown immunosuppressive potential, reducing inflammatory cytokine production and costimulatory ligands in the tissue (82). This highlights the strength of identifying specific CD8+ T-cell subsets from both conventional and single-cell approaches that can be targeted therapeutically to reduce inflammation.

Finally, following the success of B-cell depletion monoclonal antibody therapies in SLE, a direct depletion of CD8+ T-cells using monoclonal antibodies could be a future possibility for reducing inflammation in active JSLE. These therapeutic strategies have already shown promise in suppressing inflammatory damage in several experimental autoimmune models (83–86).

## Conclusions

Investigating pathogenic mechanisms of disease in JSLE addresses many unmet needs. A key advantage is that successful translation of basic research to clinical implications will hopefully result in better disease outcomes for children and young people which has significant societal implications. With this respect, JSLE patients are younger and likely have a shorter disease duration at inclusion in clinical studies, enabling the investigation of early pathogenic mechanisms associated with the disease. Disease onset in JSLE is usually more severe leading to early diagnosis and hopefully better patient stratification based on disease and therapeutic burden, as well as damage, which can mitigate against heterogeneity to immunological research, particularly when studying multi-omic data (87). There are, however, several challenges of JSLE research, including the limitation of smaller cohorts, as only 15–20% of all SLE patients have childhood onset. This makes addressing both global patient differences and heterogeneity between patients harder to explore. For younger patients, there are also more ethical issues surrounding their involvement in research, from blood collection to limited clinical trial options to test new therapies, also adding limitations to basic research and translational prospects. Larger multi-center cohort studies with long-term follow-up and bio-banked samples can answer some of the research priorities we have in this understudied patient population. Finally, more severe inflammation and organ damage in JSLE, compared with adult patients (3), suggests that we cannot assume that the underlying pathogenic mechanisms are similar across age. With this respect, more JSLE-specific research and clinical trials are required to justify the specific approval and use of therapeutics in JSLE.

Despite these challenges, the future is bright for JSLE research. Modern and sophisticated analysis and single-cell techniques now allow researchers to maximize the use of rare biological samples and investigate heterogeneity for personalized and more targeted approaches (29). With the growing evidence from both clinic and basic research studies, there is now a much-improved recognition of JSLE as an individual subset of SLE, which will hopefully improve research funding and progress, as well as increase recruitment to investigational clinical trials to address the unmet needs of this patient group. This will hopefully promote better education within this younger population about their disease and management to ultimately improve long-term quality-of-life and disease outcomes.

Here we provide strong evidence that CD8+ T-cells play a dominant pathogenic role in: (1) development; (2) severity; (3) long-term prognosis of JSLE, as well as; (4) a key association with cardiovascular co-morbidities through the acceleration of atherosclerosis (Figure 1). CD8+ T-cells should become a key target focus for new therapeutics and will be an important tool for patient stratification. Whilst it is likely that a combination of both increased CD8+ T-cell numbers and their more proinflammatory phenotype contribute to JSLE pathogenesis and outcomes, we propose that introduction of a peripheral blood T-cell immunophenotyping panel to clinical research in large cohort of patients to account for patient heterogeneity could be of huge benefit to treatment strategies and advance the understanding of JSLE pathogenesis. We advocate that creating an immune atlas for JSLE characterization in relation to organ involvement, disease activity/damage and medication, particularly focused on CD8+T cell populations, which are key drivers of peripheral blood immune abnormalities in JSLE, will help define distinct molecular signatures associated with various disease pathotypes and states. As immune phenotypic studies are improving in JSLE using new technologies, a focus on the mechanisms of CD8+ T-cell inflammation and loss of tolerance in disease will hopefully emerge. Together, this will help to direct both current and new therapies toward a more targeted and personalized approach, translating basic research for the benefit of patients from a young age.

## Author Contributions

GR performed the literature review and wrote the first draft of the manuscript. All authors reviewed the manuscript and approved the final version.

## Funding

This work was supported by a Versus Arthritis Career Development Fellowship (22856), as well as grants from the NIHR UCLH Biomedical Research Center grant BRC772/III/EJ/101350, BRC773/III/CC/101350, Lupus UK and The Rosetrees Trust (M409) and was performed within the Center for Adolescent Rheumatology Versus Arthritis at UCL. UCLH and GOSH supported by grants from Versus Arthritis (21593 and 20164), GOSCC, and the NIHR-Biomedical Research Centers at both GOSH and UCLH.

## Author Disclaimer

The views expressed are those of the authors and not necessarily those of the NHS, the NIHR or the Department of Health.

## Conflict of Interest

The authors declare that the research was conducted in the absence of any commercial or financial relationships that could be construed as a potential conflict of interest.

## Publisher's Note

All claims expressed in this article are solely those of the authors and do not necessarily represent those of their affiliated organizations, or those of the publisher, the editors and the reviewers. Any product that may be evaluated in this article, or claim that may be made by its manufacturer, is not guaranteed or endorsed by the publisher.
